# ALA-RDT in GBM: protocol of the phase I/II dose escalation trial of radiodynamic therapy with 5-Aminolevulinic acid in patients with recurrent glioblastoma

**DOI:** 10.1186/s13014-024-02408-7

**Published:** 2024-01-22

**Authors:** Niklas Benedikt Pepper, Hans Theodor Eich, Michael Müther, Michael Oertel, Stephan Rehn, Dorothee Cäcilia Spille, Walter Stummer

**Affiliations:** 1 Department of Radiation Oncology, University Hospital of Münster, Albert-Schweitzer-Campus 1, Building A1, 48149 Münster, Germany; 2 Department of Neurosurgery, University Hospital of Münster, Albert-Schweitzer-Campus 1, Building A1, 48149 Münster, Germany

**Keywords:** Glioblastoma, 5-ALA, Radiodynamic therapy, Radiation therapy, Radiosensitizer, Recurrent glioblastoma

## Abstract

**Background:**

Despite improvements in surgical as well as adjuvant therapies over the last decades, the prognosis for patients with glioblastoma remains poor. Five-Aminolevulinic acid (5-ALA) induced porphyrins are already used for fluorescence-guided resection and as photosensitizer for photodynamic therapy. New findings reveal their potential use as sensitizing agents in combination with ionizing radiation.

**Methods:**

We initiated a phase I/II dose escalation study, treating patients with recurrence of glioblastoma with oral 5-ALA concurrent to radiotherapy (RT). This prospective single-center study based in the University Hospital Münster aims to recruit 30 patients over 18 years of age with histologically verified recurrence of supratentorial glioblastoma in good performance status (KPS ≥ 60). Following a 3 + 3 dose-escalation design, patients having undergone re-resection will receive a 36 Gy RT including radiodynamic therapy fractions (RDT). RDT constitutes of oral administration of 5-ALA before the irradiation session. Two cohorts will additionally receive two fractions of neoadjuvant treatment three and two days before surgery. To determine the maximum tolerated dose of repeated 5-ALA-administration, the number of RDT-fractions will increase, starting with one to a maximum of eight fractions, while closely monitoring for safety and toxicity. Follow-up will be performed at two and five months after treatment. Primary endpoint will be the maximum tolerated dose (MTD) of repeated ALA-administration, secondary endpoints are event-free-, progression-free-, and overall-survival. Additionally, 5-ALA metabolites and radiobiological markers will be analysed throughout the course of therapy and tissue effects after neoadjuvant treatment will be determined in resected tissue. This protocol is in accordance with the SPIRIT guidelines for clinical trial protocols.

**Discussion:**

This is the protocol of the ALA-RDT in GBM-study, the first-in-man evaluation of repeated administration of 5-ALA as a radiosensitizer for treatment of recurrent glioblastoma.

**Trial registration:**

This study was approved by the local ethics committee of the Medical Association of Westphalia-Lippe and the University of Münster on 12.10.2022, the German federal institute for Drugs and medical devices on 13.10.2022 and the federal office for radiation protection on 29.08.2022. This trial was registered on the public European EudraCT database (EudraCT-No.: 2021-004631-92) and is registered under www.cliniclatrials.gov (Identifier: NCT05590689).

## Background

Glioblastoma multiforme is the most common malignant brain tumor in adults. Infamous for its poor prognosis, treatment of this highly lethal, primary brain malignancy traditionally includes resective surgery (if possible) and postoperative chemoradiation. Based on the findings of Stupp et al., the current standard of adjuvant therapy is 60 Gy of fractioned radiation treatment (RT) to a locally extended tumor site with concurrent and adjuvant application of oral temozolomide [1]. Modern radiotherapy benefits from technical and conceptual innovations like image-guided and intensity modulated treatment.

Nevertheless, recurrence seems to be inevitable for patients with glioblastoma, mainly located in or adjacent to the initial tumor site. As of today, clear treatment standards are still missing in cases of relapse or progression [2]. Re-resection and re-irradiation are treatment options in this situation [3–7], oftentimes combined with concurrent or sequential second line chemotherapy, target therapy or monoclonal antibodies, but treatment use is oftentimes limited due to prior therapy.

Multiple study approaches have aimed to increase the effectiveness of local re-treatment and re-irradiation for recurrent glioblastoma patients, attempting to achieve a more potent, more precise, and overall more effective adjuvant treatment [8–17]. In this regard, the use of radiosensitizers as a means of improving local therapy outcome by increasing effectiveness of adjuvant RT has been explored in several forms but results mostly did not match expectations and failed to improve survival when compared to standard treatment [18]. Nevertheless, new treatment agents and regimen are evaluated constantly, based on the increasing understanding of the complex characteristics and behavior of this tumor entity [11, 19–21].

Five-Aminolevulinic acid (5-ALA), a ketone carbon amino acid, has already proven to be of significant value in the treatment of glioblastoma: increasing tumor visibility under fluorescent light, based on selective uptake and production of fluorescent and photoactive porphyrins in glioma cells, surgical resection can be improved, leading to a significant increase in survival [22]. Furthermore, its application as a photoactive substance shows potential in the context of photodynamic therapy (PDT) when exposed to light of a certain wavelength (23, 24). Unfortunately, this procedure is complex, and its scope may be limited due to the restricted light penetration of brain tissue. Recent in-vitro-studies have revealed the cytotoxic effect (similar to PDT) to also be inducible by exposure of cells containing the metabolite Protoporphyrin IX (PPIX) to ionizing radiation, resulting in increased cell damage, based on a delayed mitochondrial production of reactive oxygen-species (ROS) (25, 26). While this has been evaluated in several preclinical settings for different tumor entities [27–30], no studies involving humans have evaluated the concept of repeated application of 5-ALA during the course of fractioned RT yet. When compared to other radiosensitizing agents, 5-ALA seems to have an advantage due to its tumor-selective behavior deriving from its active uptake into glioma cells via specific transporters [31–33] after extravasation and distribution along the infiltration zone by local edema [34]. Combined with the overall low toxicity (known from its well implemented use in resective surgery) and easy handling via oral application, 5-ALA appears to be a strong contender to improve therapy effectiveness without immoderate risk of increasing side effects. In this article, a description of our protocol for the recently initiated “ALA-RDT in GBM” 3 + 3 dose escalation trial is given, aiming to further explore the potential of this promising combination.

## Methods

### Trial design, setting and recruitment

This is a prospective, single-armed, single center phase I/II dose escalation-study, based in the University Hospital of Münster, Germany. The aim of the study is to determine the maximum tolerated dose (MTD) and safety of repeated application of oral 5-ALA as a radiosensitizer during (neo)adjuvant radiotherapy for the treatment of glioblastoma recurrence. All patients will receive 36 Gy adjuvant RT in 20 fractions. An increasing number of RT-fractions will be conducted as radiodynamic therapy (RDT), which will consist of 1.8 Gy-irradiation 7–9 h after oral application of 20 mg/kg bodyweight 5-ALA in a tap-water-solution.

Recruitment will start in cohort 0 with one RDT-fraction and patients will be allocated consecutively to this and the following cohorts, which will be numbered sequentially from 0 to 8. Each cohort encompasses three initial patients being expanded by additional three participants in case of a dose limiting toxicity (DLT), following a 3 + 3-design. With increasing cohort number, the quantity of RDT-fractions will be increased by one with a maximum of eight adjuvant applications in cohort 8. Additionally, in cohorts 0 and 1, patients will receive two neoadjuvant RT-fractions (in case of cohort 1, the first fraction will be conducted as RDT). In these cohorts, a decisive histopathological analysis of the resected tissue will account for changes induced by neoadjuvant therapy. MDT is considered to be reached in case of DLT in two or more patients on the same dose level. A study continuation with the next dose-level will be evaluated and approved by the data safety and monitoring board (DSMB) after a six-week follow-up of the last RT-fraction of the last patient within a cohort. The study started recruitment with the first patient in Q1 2023 with an estimated study duration of 30 months.

### Study population

26 patients will be recruited with additional 15–20% drop-outs, cumulating in approximately 30 patients. End of study is defined as the last patient’s last visit (six-month follow-up). For each drop-out, a new patient will be entered into the study at the same dose level.

### Inclusion criteria

1: Written patient consent after comprehensive information.

2: Age ≥ 18 years.

3: Recurrence of supratentorial glioblastoma after initial resection and adjuvant therapy.

(e.g. radio-chemotherapy, targeted therapies, antiangiogenic therapies as determined.

by the tumor board) (with planned second resection cohort 0 and 1), second or third.

recurrences permitted.

4: Clinically indicated further radiotherapy as per decision of the tumor board as part of.

therapy for recurrence.

5: Histological verification of recurrent glioblastoma independent of methylated MGMT.

promotor status when alkylating chemotherapy failed at this time.

6: Karnofsky Performance Score ≥ 60.

7: For all patients of reproductive potential: Willingness to apply highly.

effective contraception (Pearl index < 1) during the entire study (and for at least 6.

months after the last application of 5-ALA)

8: Pre-menopausal female patients with childbearing potential: a negative serum pregnancy test must be obtained max. 72 h prior to treatment start.

9: Adequate liver function: bilirubin < 1.5 times above upper limit of normal range (ULN), alanine transaminase (ALT/SGPT) and aspartate transaminase (AST/SGOT) < 3 times ULN. In the case of documented or suspected Gilbert’s disease bilirubin < 3 times ULN.

10: Adequate renal function: creatinine < 3 times above ULN; eGFR >/= 60 ml/min, Blood clotting: INR/Quick/PT and PTT within acceptable limits according to the investigator.

### Exclusion criteria

1: Patient unable to undergo imaging by MRI, PET or contrast-enhanced CT for whatever reason (e.g. pacemaker).

2: Pregnant or breastfeeding women.

3: Past medical history of diseases with poor prognosis, e.g., severe coronary heart disease, heart failure (NYHA III/IV), severe and poorly controlled diabetes, immune deficiency, residual deficits after stroke, severe mental retardation or other serious concomitant systemic disorders incompatible with the study (at the discretion of the investigator).

4: Any active infection (at the discretion of the investigator).

5: Hypersensitivity against porphyrins.

6: Known diagnosis of porphyria.

7: Current participation in another clinical trial with therapeutic intervention or use of any other therapeutic interventional agent other than the standard therapy since diagnosis of glioblastoma.

8: Known intolerance to study medication.

9: Pre-treatment with other potentially phototoxic or photosensitizing substances (e.g. tetracyclines, sulfonamides, fluoroquinolones, hypericin extracts, products containing St. John’s wort) during the two weeks preceding RDT.

### Treatment procedures

#### Radiation treatment

The indication for second irradiation will be discussed and decided in the multidisciplinary tumor board before the screening process. Adjuvant radiotherapy will consist of fractioned photon irradiation, delivering a total dose of 36 Gy in 20 fractions, with five fractions applied per week (Monday through Friday). In cohorts 0 and 1, patients will receive two additional fractions of 1.8 Gy as neoadjuvant treatment, resulting in a cumulative dose of 39.6 Gy. Target definition and dose prescription will be according to ICRU 83 (for intensity-modulated radiotherapy) and planned with dedicated computed tomography (CT), pre- and postoperative contrast-enhanced MRI and three-dimensional planning systems. Gross tumor volume (GTV) will be defined in the pre- and postoperative MRI as the extend of the contrast-enhancing T1-hyperintense tumor site, the clinical target volume (CTV) will consist of a 1 cm margin around the GTV, also considering additional imaging (if available), respecting anatomical borders, and considering pre-treatment dose distributions. The planning target volume (PTV) will be defined as a 0.3–0.5 cm margin around the CTV. Dose constraints will be considered according to QUANTEC-recommendations. Intensity-modulated radiotherapy will be delivered with linear accelerators with a beam energy of 6 MV or 6 MV FFF, quality assurance will be performed by means of individual case reviews and image-guidance via frequent cone beam CT.

Preoperative irradiation will be performed in two fractions; one fraction daily, three and two days before the scheduled operative resection. Postoperative irradiation will begin after clinical recovery two to six weeks (as clinically indicated) after surgery.

#### Drug administration

Oral 5-Aminolevulinic acid will be applied on RDT-treatment days as a solution in tap water with a dose of 20 mg/kg bodyweight between seven and nine hours prior to 1.8 Gy-irradiation. Additionally, patients will receive 5-ALA four hours prior to induction of anesthesia for surgery. Use of additional concomitant chemo- or immunotherapy is prohibited during trial participation.

#### Blood and tissue samples

Tissue sampling and evaluation of morphological changes in brain tissue will be performed on cohorts 0 and 1 after neoadjuvant treatment. These analyses will therefore include tissue changes after sole RT (cohort 0) as well as RDT (cohort 1), based on parameters like Caspase-3, IBA1, H&E, EvG, P53, Ki67 and gammaH2AX. Blood samples will be drawn for safety clearance as part of toxicity assessment and to monitor changes of ALA-metabolites in serum (PPIX) before every application of 5-ALA. Additional blood samples for analysis of concentration changes in radiobiological markers (including markers like IL-6, IL-8, TNF-Alpha and VEGF) will be drawn during neoadjuvant treatment (day − 3 and day − 2) and at the beginning (day 1 and day 2) and end (day 20) of adjuvant treatment.

Concentration changes of 5-ALA-metabolites in urine samples will be evaluated during neoadjuvant treatment (day − 3) and surgery (day 0) for cohorts 0 and 1 and at the beginning (day 1) and first follow-up for all study participants.

### Treatment days

Tables 1 and 2 illustrate the treatment schedule and sequence of enrollment, follow-up and interventions, based on the SPIRIT-guidelines for publication of trial protocols.

**Table 1:** Schedule for Screening and follow-up of the ALA-RDT in GBM study

	Screening for cohorts 0 and 1	Screening for cohorts 2 to 8	Follow-Up 1 (all patients)	Follow-Up 2 (all patients)
Day	-7 to -4 before surgery	-14 to -2 before first RDT	12 weeks after firstadjuvant RT	6 months after firstadjuvant RT
**Informed consent**:	X	X		
**In- / Exclusion criteria**:	X	X		
**MRI**:	MRI outside screening period used for diagnosis and as baseline value	MRI up to 72 h after surgery, performed outside study protocol Screening period used for diagnosis and baseline	X	X
**Medical and neurological examination**	X	X	X	X
**Laboratory panel**	X	X	X	X
**Medical / surgical history**	X	X		

**Table 2:** Schedule for procedures of the ALA-RDT in GBM study

		Neoadj. RT		Surgery		Adjuvant RT (beginning 2–6 weeks as clinically indicated after surgery)																				
		*(pre)OP*					*1. week*					*2. week*					*3. week*					*4. week*				
		*Treatment Day*																								
**Cohort**	**Intervention**	*-3*	*-2*		*0*		*1*	*2*	*3*	*4*	*5*	*6*	*7*	*8*	*9*	10	*11*	*12*	*13*	*14*	*15*	*16*	*17*	*18*	*19*	*20*
**0**	RT with 1.8 Gy	X	X				X	X	X	X	X	X	X	X	X	X	X	X	X	X	X	X	X	X	X	X
	Surgery of rGBM				X																					
	5-ALA before RT (RDT)						X																			
**1**	RT with 1.8 Gy	X	X				X	X	X	X	X	X	X	X	X	X	X	X	X	X	X	X	X	X	X	X
	Surgery of rGBM				X																					
	5-ALA before RT (RDT)	X																								
**2**	RT with 1.8 Gy						X	X	X	X	X	X	X	X	X	X	X	X	X	X	X	X	X	X	X	X
	Surgery of rGBM				X																					
	5-ALA before RT (RDT)						X					X														
**3**	RT with 1.8 Gy						X	X	X	X	X	X	X	X	X	X	X	X	X	X	X	X	X	X	X	X
	Surgery of rGBM				X																					
	5-ALA before RT (RDT)						X					X					X									
**4**	RT with 1.8 Gy						X	X	X	X	X	X	X	X	X	X	X	X	X	X	X	X	X	X	X	X
	Surgery of rGBM				X																					
	5-ALA before RT (RDT)						X					X					X					X				
**5**	RT with 1.8 Gy						X	X	X	X	X	X	X	X	X	X	X	X	X	X	X	X	X	X	X	X
	Surgery of rGBM				X																					
	5-ALA before RT (RDT)						X			X		X					X					X				
**6**	RT with 1.8 Gy						X	X	X	X	X	X	X	X	X	X	X	X	X	X	X	X	X	X	X	X
	Surgery of rGBM				X																					
	5-ALA before RT (RDT)						X			X		X			X		X					X				
**7**	RT with 1.8 Gy						X	X	X	X	X	X	X	X	X	X	X	X	X	X	X	X	X	X	X	X
	Surgery of rGBM				X																					
	5-ALA before RT (RDT)						X			X		X			X		X			X		X				
***8***	RT with 1.8 Gy						X	X	X	X	X	X	X	X	X	X	X	X	X	X	X	X	X	X	X	X
	Surgery of rGBM				X																					
	5-ALA before RT (RDT)						X			X		X			X		X			X		X			X	

### Toxicity assessment

All patients will be hospitalized for the course of treatment procedure and receive daily AE/SAE-monitoring. Physical and neurological examinations will be performed routinely on a weekly basis and laboratory panels for toxicological safety will be drawn before every RDT-fraction, but also at least weekly. Toxicity will be monitored daily and classified according to CTCAE Vers. 5.0. All serious adverse events will be immediately reported to the Data Safety Monitoring Board.

### Follow-Up

All patients will receive a six-week follow-up after the end of radiotherapy to evaluate sequential side effects of repeated administration of 5-ALA. Additional follow-ups, including MRI-scans, will be performed twelve weeks and six months after the beginning of adjuvant treatment.

### End-points

Primary: To determine the maximum tolerated dose and safety of (neo)adjuvant RDT. MTD is defined as maximum dose level before two or more patients suffer from DLT.

Secondary: To evaluate six-months overall survival rate (OSR), six-months progression-free survival rate (PFSR) and six-months event-free survival rate (EFSR), as well as histological tissue changes after neoadjuvant treatment. OSR, PFSR and EFSR will be determined from study inclusion to the second follow-up, six months after the beginning of adjuvant treatment.

Exploratory: To explore concentration changes of 5-ALA metabolites in urine samples and changes of radiobiological marker concentration in blood serum samples.

### Statistical analysis

Statistical analyses will include “intention-to-treat”-evaluation for all cohorts’ primary and secondary endpoints and “per-protocol”-evaluation for explorative endpoints. “Per protocol” is defined as administration of RT- and 5-ALA-applications according to prescribed dose. Descriptive measures for all variables will be given. Rates of adverse events will include all events qualifying for CTCAE grade 3 or greater and the final DLT-rate will be estimated by use of the Bayesian Time-to-Event Continual Reassessment Method (Tite-CRM). Survival parameters will be analyzed using the Kaplan-Meier method and two-sided log-rank tests for comparative analyses between defined subgroups. Cox regression testing will estimate impact of risk factors (e.g. age, KPS, gender) on these parameters.

All data will be compared to historical data using the Mann-Whitney U test, Fisher’s exact test, or Chi² test.

## Discussion

The treatment of recurrent glioblastoma lacks clear standards with re-resection, re-irradiation and systemtic agents being the pillars of therapy. A recent phase II trial (RTOG1205, [35]) has demonstrated a PFS-benefit when combining salvage therapy (in the form of bevacicumab) with re-RT. Nevertheless, second treatment of all modalities oftentimes suffers from limitations and improvements of therapy effectiveness are needed. Based on the well-established use of 5-ALA for surgery of glioblastoma [22, 36–38] and promising preclinical data [27–30], this study aims to answer the question if the addition of repeated applications of oral 5-ALA during re-irradiation is safe and may be beneficial towards survival parameters. While several other agents did not result in improvement of outcome [18], the use of 5-ALA as a radiosensitizer is especially promising because of the selective uptake in glioma tumor cells, sparing normal brain tissue [34, 39]. Moreover, 5-ALA is easy to apply and generally tolerated well, for example in the context of surgery and PDT. We chose to design this trial in the setting of recurrent glioblastoma, based on the high need for effective salvage treatment options in this patient cohort and the lack of a clear treatment standard. In case of positive results, further evaluation as an addition to standard first line treatment may be attempted.

However, no data is available regarding consecutive applications of the drug yet. In the context of brain tumor surgery single doses of up to 60 mg/kg have previously been reported to be well tolerated [40], so that a dose escalation trial using 20 mg/kg increments appears to be justified.

Despite that fact, cumulative effects resulting in hepatotoxicity or increased photosensitivity from repetitive applications cannot be ruled out. The blood and urine samples collected during the study aim to understand instances of drug- or metabolite-accumulation and to prevent them in the future.

Another limitation might arise from uncertainties regarding tissue reactions to the novel concept of ALA-RDT: increased occurrence of radiation side effects like brain tissue necrosis or local brain edema, due to additive interactions, might limit patients’ quality of life and even prognosis. Therefore, close monitoring of the patients’ clinical status and signs for neurological side effects are vital, as well as a careful approach regarding daily fraction-dose (1.8 Gy). We expect any undue tissue reactions such as edema to be self-limiting and that corticosteroids will help in ameliorating symptoms. Nevertheless, the inclusion of neoadjuvant treatment (and RDT) in the first cohorts also serves as an additional fail-save to detect excessive reactions in surrounding brain tissue early on.

Additionally, while the concept of radiosensitization has been established for different wavelengths in laboratory setups (25–26, 41), the interaction with 6MV-photons, commonly used in cerebral irradiation, remains uncertain.

In conclusion, we consider that the results of this single-center, first-in-human dose-escalation study could provide a valuable tool in the therapy of patients with glioblastoma and lead to a new era of increased effectiveness of radiotherapy in this highly aggressive tumor entity.

## Data Availability

No datasets were generated or analysed during the current study.

## Abbreviations

5-ALA: 5-aminolevulinic acid
DSMB: Data safety and monitoring board
EFSR: Event-free survival rate
EvG: Elastica-van-Gieson stain
H&E: Hematoxylin and eosin stain
IL-6/IL-8: Interleukin 6/8KPS:Karnofsky Performance Status
MGMT: O-6-methylguanine-DNA methyltransferase
OSR: Overall-survival-rate
PFSR: Progression-free survival rate
PDT: Photodynamic therapyRDT:Radiodynamic therapy
ROS: Reactive Oxygen Species
RT: Radiotherapy
TNF-Alpha: Tumor necrosis factor alpha
VEGF: Vascular endothelial growth factor

## References

[1] Stupp R, Mason WP, van den Bent MJ (2005). Radiotherapy plus concomitant and adjuvant temozolomide for glioblastoma. N Engl J Med.

[2] Weller M, Tabatabai G, Kästner B (2015). MGMT promoter methylation is a strong prognostic biomarker for benefit from dose-intensified Temozolomide Rechallenge in Progressive Glioblastoma: the DIRECTOR trial. Clin Cancer Res.

[3] Quick J, Gessler F, Dützmann S, Hattingen E, Harter PN, Weise LM, Franz K, Seifert V, Senft C (2014). Benefit of tumor resection for recurrent glioblastoma. J Neurooncol.

[4] Hennessy MA, Coyne ZL, O’Halloran PJ, Mullally W, Dablouk M, MacNally S, Morris PG (2022). Prognostic factors influencing survival following re-resection for isocitrate dehydrogenase (IDH) -wildtype glioblastoma multiforme - data from a national neuro-oncology registry. J Clin Neurosci.

[5] Baehr A, Trog D, Oertel M, Welsch S, Kröger K, Grauer O, Haverkamp U, Eich HT. Re-irradiation for recurrent glioblastoma multiforme: a critical comparison of different concepts. Strahlenther Onkol. 2020;196(5):457–464. 10.1007/s00066-020-01585-0. Epub 2020 Feb 3. PMID: 32016497. 10.1007/s00066-020-01585-0 32016497

[6] Kazmi F, Soon YY, Leong YH, Koh WY, Vellayappan B (2019). Re-irradiation for recurrent glioblastoma (GBM): a systematic review and meta-analysis. J Neurooncol.

[7] Combs SE, Bischof M, Welzel T, Hof H, Oertel S, Debus J, Schulz-Ertner D (2008). Radiochemotherapy with temozolomide as re-irradiation using high precision fractionated stereotactic radiotherapy (FSRT) in patients with recurrent gliomas. J Neurooncol.

[8] Straube C, Scherb H, Gempt J, Kirschke J, Zimmer C, Schmidt-Graf F, Meyer B, Combs SE (2018). Adjuvant stereotactic fractionated radiotherapy to the resection cavity in recurrent glioblastoma - the GlioCave study (NOA 17 - ARO 2016/3 - DKTK ROG trial). BMC Cancer.

[9] Oehlke O, Mix M, Graf E, Schimek-Jasch T, Nestle U, Götz I, Schneider-Fuchs S, Weyerbrock A, Mader I, Baumert BG, Short SC, Meyer PT, Weber WA, Grosu AL (2016). Amino-acid PET versus MRI guided re-irradiation in patients with recurrent glioblastoma multiforme (GLIAA) - protocol of a randomized phase II trial (NOA 10/ARO 2013-1). BMC Cancer.

[10] National Library of Medicine (U.S.). (2020, July). Stereotactical photodynamic therapy with 5-aminolevulinic acid (Gliolan®) in recurrent glioblastoma (NOA11). Identifier NCT04469699. https://clinicaltrials.gov/ct2/show/results/NCT04469699.

[11] Zeyen T, Potthoff AL, Nemeth R, Heiland DH, Burger MC, Steinbach JP, Hau P, Tabatabai G, Glas M, Schlegel U, Grauer O, Krex D, Schnell O, Goldbrunner R, Sabel M, Thon N, Delev D, Clusmann H, Seidel C, Güresir E, Schmid M, Schuss P, Giordano FA, Radbruch A, Becker A, Weller J, Schaub C, Vatter H, Schilling J, Winkler F, Herrlinger U, Schneider M (2022). Phase I/II trial of meclofenamate in progressive MGMT-methylated glioblastoma under temozolomide second-line therapy-the MecMeth/NOA-24 trial. Trials.

[12] Sahebjam S, Forsyth PA, Tran ND, Arrington JA, Macaulay R, Etame AB, Walko CM, Boyle T, Peguero EN, Jaglal M, Mokhtari S, Enderling H, Raghunand N, Gatewood T, Long W, Dzierzeski JL, Evernden B, Robinson T, Wicklund MC, Kim S, Thompson ZJ, Chen DT, Chinnaiyan P, Yu HM (2021). Hypofractionated stereotactic re-irradiation with pembrolizumab and bevacizumab in patients with recurrent high-grade gliomas: results from a phase I study. Neuro Oncol.

[13] Lederman G, Wronski M, Arbit E, Odaimi M, Wertheim S, Lombardi E, Wrzolek M. Treatment of recurrent glioblastoma multiforme using fractionated stereotactic radiosurgery and concurrent paclitaxel. Am J Clin Oncol. 2000;23(2):155-9. 10.1097/00000421-200004000-00010. PMID: 10776976. 10.1097/00000421-200004000-00010 10776976

[14] Balducci M, Diletto B, Chiesa S, D’Agostino GR, Gambacorta MA, Ferro M, Colosimo C, Maira G, Anile C, Valentini V (2014). Low-dose fractionated radiotherapy and concomitant chemotherapy for recurrent or progressive glioblastoma: final report of a pilot study. Strahlenther Onkol.

[15] Guan Y, Xiong J, Pan M, Shi W, Li J, Zhu H, Gong X, Li C, Mei G, Liu X, Pan L, Dai J, Wang Y, Wang E, Wang X (2021). Safety and efficacy of hypofractionated stereotactic radiosurgery for high-grade gliomas at first recurrence: a single-center experience. BMC Cancer.

[16] Song A, Andrews DW, Werner-Wasik M, Kim L, Glass J, Bar-Ad V, Evans JJ, Farrell CJ, Judy KD, Daskalakis C, Zhan T, Shi W. Phase I trial of alisertib with concurrent fractionated stereotactic re-irradiation for recurrent high grade gliomas. Radiother Oncol. 2019;132:135–141. doi: 10.1016/j.radonc.2018.12.019. Epub 2019 Jan 4. PMID: 30825962. 10.1016/j.radonc.2018.12.019 30825962

[17] de Groot JF, Lamborn KR, Chang SM, Gilbert MR, Cloughesy TF, Aldape K, Yao J, Jackson EF, Lieberman F, Robins HI, Mehta MP, Lassman AB, Deangelis LM, Yung WK, Chen A, Prados MD, Wen PY (2011). Phase II study of aflibercept in recurrent malignant glioma: a north American brain Tumor Consortium study. J Clin Oncol.

[18] Pepper NB, Stummer W, Hans HT (2022). The use of radiosensitizing agents in the therapy of glioblastoma multiforme—a comprehensive review. Strahlenther Onkol.

[19] National Library of Medicine (U.S.). (2019, October). Glioblastoma Treatment with Irradiation and Olaptesed Pegol (NOX-A12) in MGMT Unmethylated patients (GLORIA). Identifier NCT04121455. https://clinicaltrials.gov/ct2/show/NCT04121455.

[20] So JS, Kim H, Han KS (2021). Mechanisms of Invasion in Glioblastoma: Extracellular Matrix, Ca2 + signaling, and Glutamate. Front Cell Neurosci.

[21] Jung E, Osswald M, Ratliff M, Dogan H, Xie R, Weil S, Hoffmann DC, Kurz FT, Kessler T, Heiland S, von Deimling A, Sahm F, Wick W, Winkler F (2021). Tumor cell plasticity, heterogeneity, and resistance in crucial microenvironmental niches in glioma. Nat Commun.

[22] Stummer W, Pichlmeier U, Meinel T, Wiestler OD, Zanella F, Reulen HJ (2006). Fluorescence-guided surgery with 5-aminolevulinic acid for resection of malignant glioma: a randomised controlled multicentre phase III trial. Lancet Oncol.

[23] Beck TJ, Kreth FW, Beyer W (2007). Interstitial photodynamic therapy of nonresectable malignant glioma recurrences using 5-aminolevulinic acid induced protoporphyrin IX. Lasers Surg Med.

[24] Mahmoudi K, Garvey KL, Bouras A (2019). 5-aminolevulinic acid photodynamic therapy for the treatment of high-grade gliomas. J Neurooncol.

[25] Ueta K, Yamamoto J, Tanaka T, Nakano Y, Kitagawa T, Nishizawa S (2017). 5-Aminolevulinic acid enhances mitochondrial stress upon ionizing irradiation exposure and increases delayed production of reactive oxygen species and cell death in glioma cells. Int J Mol Med.

[26] Kitagawa T, Yamamoto J, Tanaka T, Nakano Y, Akiba D, Ueta K, Nishizawa S (2015). 5-Aminolevulinic acid strongly enhances delayed intracellular production of reactive oxygen species (ROS) generated by ionizing irradiation: quantitative analyses and visualization of intracellular ROS production in glioma cells in vitro. Oncol Rep.

[27] Yamamoto J, Ogura SI, Shimajiri S (2015). 5-Aminolevulinic acid-induced protoporphyrin IX with multi-doseionizing irradiation enhances host antitumor response and strongly inhibits tumor growth in experimental glioma in vivo. Mol Med Rep.

[28] Wang B, Cvetkovic D, Gupta R, Chen L, Ma CMC, Zhang Q, Zeng J (2015). Radiation therapy combined with 5-aminolevulinic acid: a preliminary study with an in vivo mouse model implanted with human PC-3 tumor cells. Int J Radiat Oncol Biol Phys.

[29] Owari T, Tanaka N, Nakai Y (2022). 5-Aminolevulinic acid overcomes hypoxia-induced radiation resistance by enhancing mitochondrial reactive oxygen species production in prostate cancer cells [published online ahead of print, 2022 Apr 1]. Br J Cancer.

[30] Takahashi J, Murakami M, Mori T, Iwahashi H. Verification of radiodynamic therapy by medical linear accelerator using a mouse melanoma tumor model. *Sci Rep*. 2018;8(1):2728. Published 2018 Feb 9. 10.1038/s41598-018-21152-z. 10.1038/s41598-018-21152-z PMC5807383 29426920

[31] Zimmermann M, Stan AC (2010). PepT2 transporter protein expression in human neoplastic glial cells and mediation of fluorescently tagged dipeptide derivative beta-ala-lys-nepsilon-7-amino-4-methyl-coumarin-3-acetic acid accumulation. J Neurosurg.

[32] Hagiya Y, Fukuhara H, Matsumoto K (2013). Expression levels of PEPT1 and ABCG2 play key roles in 5-aminolevulinic acid (ALA)-induced tumor-specific protoporphyrin IX (PpIX) accumulation in bladder cancer. Photodiagnosis Photodyn Ther.

[33] Tran TT, Mu A, Adachi Y, Adachi Y, Taketani S (2014). Neurotransmitter transporter family including SLC6A6 and SLC6A13 contributes to the 5-aminolevulinic acid (ALA)-induced accumulation of protoporphyrin IX and photodamage, through uptake of ALA by cancerous cells. Photochem Photobiol.

[34] Stepp H, Stummer W (2018). 5-ALA in the management of malignant glioma. Lasers Surg Med.

[35] Tsien CI, Pugh SL, Dicker AP, Raizer JJ, Matuszak MM, Lallana EC, Huang J, Algan O, Deb N, Portelance L, Villano JL, Hamm JT, Oh KS, Ali AN, Kim MM, Lindhorst SM, Mehta MP. NRG Oncology/RTOG1205: a randomized phase II trial of concurrent Bevacizumab and Reirradiation Versus Bevacizumab alone as treatment for recurrent glioblastoma. J Clin Oncol. 2023;41(6):1285–95. Epub 2022 Oct 19. PMID: 36260832; PMCID: PMC9940937. 10.1200/JCO.22.00164 PMC9940937 36260832

[36] Díez Valle R, Hadjipanayis CG, Stummer W. Established and emerging uses of 5-ALA in the brain: an overview. J Neurooncol. 2019;141(3):487–494. 10.1007/s11060-018-03087-7. Epub 2019 Jan 3. PMID: 30607705. 10.1007/s11060-018-03087-7 30607705

[37] Hadjipanayis CG, Stummer W (2019). 5-ALA and FDA approval for glioma surgery. J Neurooncol.

[38] Picart T, Pallud J, Berthiller J, Dumot C, Berhouma M, Ducray F, Armoiry X, Margier J, Guerre P, Varlet P, Meyronet D, Metellus P, Guyotat J, Members of RESECT study group:.;. Use of 5-ALA fluorescence-guided surgery versus white-light conventional microsurgery for the resection of newly diagnosed glioblastomas (RESECT study): a French multicenter randomized phase III study. J Neurosurg 2023 Oct 13:1–14. doi: 10.3171/2023.7.JNS231170. Epub ahead of print. PMID: 37856381. 10.3171/2023.7.JNS231170 37856381

[39] Traylor JI, Pernik MN, Sternisha AC, McBrayer SK, Abdullah KG (2021). Molecular and metabolic mechanisms underlying selective 5-Aminolevulinic Acid-Induced fluorescence in Gliomas. Cancers (Basel).

[40] Cozzens JW, Lokaitis BC, Moore BE, Amin DV, Espinosa JA, MacGregor M, Michael AP, Jones BA. A Phase 1 Dose-Escalation Study of Oral 5-Aminolevulinic Acid in Adult Patients Undergoing Resection of a Newly Diagnosed or Recurrent High-Grade Glioma. Neurosurgery. 2017;81(1):46–55. 10.1093/neuros/nyw182. PMID: 28498936. 10.1093/neuros/nyw182 28498936

[41] Panetta JV, Cvetkovic D, Chen X, Chen L, Ma CC. Radiodynamic therapy using 15-MV radiation combined with 5-aminolevulinic acid and carbamide peroxide for prostate cancer in vivo. Phys Med Biol. 2020;65(16):165008. Published 2020 Aug 19. 10.1088/1361-6560/ab9776. 10.1088/1361-6560/ab9776 PMC8409498 32464613

